# Prevalence and correlates of lifetime and recent HIV testing among men who have sex with men (MSM) who use mobile geo-social networking applications in Greater Tokyo

**DOI:** 10.1371/journal.pone.0209933

**Published:** 2019-01-23

**Authors:** Adam O. Hill, Benjamin R. Bavinton, Gregory Armstrong

**Affiliations:** 1 Faculty of Arts, School of Social and Political Sciences, University of Melbourne, Parkville, VIC, Australia; 2 Kirby Institute, University of New South Wales, Sydney, NSW, Australia; 3 Nossal Institute for Global Health, Melbourne School of Population and Global Health, University of Melbourne, Parkville, VIC, Australia; University of New South Wales, AUSTRALIA

## Abstract

Men who have sex with men (MSM) are disproportionately burdened by the human immunodeficiency virus (HIV), accounting for 78% of all Japanese male HIV cases in 2016. Over 30% of newly identified HIV infections in Japan are diagnosed as AIDS annually, suggesting a large proportion of people living with HIV were unaware of their own infection status. An estimated two-thirds of Japanese men who have sex with men (MSM) are not attached to the gay community, and previous studies have largely sampled gay venues, thus, previous studies have likely failed to reach many men in this population. This study therefore examined HIV testing prevalence and correlates among MSM in Greater Tokyo who use gay mobile geo-social networking applications (gay mobile apps), which have been found to increase access to MSM not traditionally accessible through venue-based surveys. Among a sample of 1657 MSM recruited through advertisements on gay mobile apps, the prevalence of lifetime and six-monthly HIV testing was 72.8% and 29.7% respectively. In multiple regression analysis, higher lifetime HIV testing was associated with older age, education, HIV knowledge, anal intercourse with regular and casual male partners, and gay venue attendance. Testing was negatively associated with regular male partner condom use, marriage, residing outside central Tokyo and having both male and female partners. These results indicated that MSM who use gay mobile apps in Greater Tokyo do not meet the CDC yearly testing recommendations for high risk populations. Considering limited HIV prevention funding in Japan for MSM, moderate lifetime and recent testing, and the large number of gay mobile app users, utilization of popular gay mobile apps to promote nearby HIV testing facilities may be an effective prevention policy to target non-community attached MSM, particularly at-risk youth and individuals at risk of sudden-onset AIDS.

## Introduction

HIV testing is an essential component of HIV prevention. HIV testing has positive effects for both HIV prevention and care, and it is well documented that people who learn they are HIV-positive take preventive action to reduce the risk of transmission to others [1], and testing is associated with a 68% efficacy for lowering condomless anal intercourse (CLAI) in meta-analysis [2]. Testing is central to the UNAIDS 90-90-90 Targets, with the first of the goals being to diagnose 90% of all HIV-positive individuals by 2020 [3]. Moreover, testing is an essential prerequisite for effective treatment as prevention (TasP) strategies, in which all HIV-positive people are treated with anti-retroviral therapy (ART) to stop onward infection to future sexual partners [4,5], and has been found highly effective at preventing HIV transmission in serodiscordant couples in both heterosexual [4] and more recently, homosexual couples [6,7]. Frequent testing also reduces the likelihood of late diagnosis and the number of acquired immunodeficiency virus (AIDS) diagnoses.

Men who have sex with men (MSM) are estimated to make up between 4.0 to 4.6% of the male population in Japan, but are disproportionately burdened by the human immunodeficiency virus (HIV) [8,9,10], accounting for 78% of all Japanese male HIV cases, though this is likely to be higher due to underreporting of MSM transmission [11]. Although AIDS has become infrequent among MSM in Western developed nations such the UK [12], it remains a substantial health burden for MSM in Japan where over 30% of newly diagnosed HIV cases were reported as AIDS in 2016 (1,011 HIV and 437 AIDS) [13]. Moreover, this figure is likely to be underreported as some small healthcare centers, where approximately 40% of Japanese HIV tests take place, do not differentiate between HIV and AIDS and report all cases as HIV [14]. Furthermore, the volume of HIV tests in Japan has reduced by 35% from their peak of 177,000 in 2008 to 118,000 in 2016 due to fewer advertising campaigns and redirection of resources from HIV prevention to AIDS care, and the subsequent closure of free testing centres [15]. Considering the network of health services providing free testing and access to treatment in Greater Tokyo, HIV testing is still infrequent compared to other countries with similar services. From 2011 to 2014, one third (32%) of patients newly diagnosed with HIV went for voluntary testing; over half (53%) were reported due to presence of other diseases, unchanged from 2000 to 2004 [16].

Previous studies among Japanese MSM have focused on the gay community-attached MSM population using traditional venue-based sampling techniques. However, an estimated two-thirds of Japanese MSM are not attached to the gay community [17], thus, previous studies have likely failed to reach many men in this population. Because gay geo-social networking applications, henceforth referred to as gay mobile apps, have users who are both attached and non-community-attached, they have been found to increase access to MSM not traditionally accessible through venue-based surveys [18–22]. Gay mobile apps enable users to use global positioning systems (GPS) in mobile phones to search through a grid of nearby users based on their proximity, and to contact and meet nearby users, enabling MSM to encounter each other more quickly and easily [22,23]. Gay mobile apps have very quickly become one of the most popular ways to arrange sex among MSM globally [24] and in Japan [25]. Although Grindr is currently the most popular gay mobile application in the world with almost 3 million active daily users [26], Japanese application use varies significantly from the West. In 2017, the most used application was 9Monster, with 300,000 Japanese users as of April 2017 [27]. While gay internet use has been extensively researched [28], there is a distinct lack of research about MSM who use gay mobile apps outside the US, Australia, and China [29]. Existing research has shown gay mobile app users to have higher numbers of sexual partners [18,30], and higher rates of HIV testing [18,29] than non-app using MSM.

This research therefore utilizes a gay mobile app based cross-sectional survey to investigate the frequency and psychosocial correlates of lifetime and recent HIV testing and future testing intention among gay mobile app using MSM. Understanding testing rates and testing intention among this hard-to-reach population of gay mobile app using MSM improves our understanding of different MSM subgroups, and in doing so enables us to better profile the Japanese HIV epidemic and potentially tailor prevention strategies for different MSM populations.

## Materials and methods

### Recruitment

Subjects were recruited using the geo-location feature of gay mobile apps, similar to methods previously used to recruit Grindr users in the US [22,31]. Popular gay mobile apps were used (e.g. jack’d, Hornet, Grindr, 9Monster) that were used in a previous similar study recruiting MSM in Japan [32], and were recommended during piloting in order to gain access to the most MSM. Gay mobile apps use geo-location to sort users by proximity, with the users closest to the researcher displayed at the top of a grid of photos with 3–4 photos per row. The researcher was positioned in Tokyo centrally, launched the most popular in Japan and randomly selected one user from each row until 50 previously uncontacted users were messaged with a link to the survey each day. Recruitment messages consisting of a greeting and short message regarding the purpose of the survey and link were sent to gay mobile app users. Messages were logged by the app chat functions, hence previously messaged users were recorded and not messaged again. The survey was conducted between November 22^nd^ 2015 to January 16^th,^ 2016, recruiting 215 valid respondents. Slow recruitment due to frequent social networking services (SNS) scamming in Japan [33], in which users are often messaged by fake accounts attempting to steal personal information, and subsequent potential recruitment bias in which many respondents would not reply, led to a change in recruitment strategy. Because an advertisement must be screened by app companies prior to its promotion in-app, this was deemed more trustworthy to potentially recruit more participants. A splash screen poster (a full-screen advertisement that appears when the user opens the app) encouraging application users to respond to the linked survey was placed on 9Monster, the most popular Japanese gay mobile app [27], for one week from January 17^th^ to January 23^rd^, 2017 to supplement response numbers. This methodology was used previously with success in the US [19,21]. The advertisement was displayed only to MSM who used gay mobile apps in Greater Tokyo. It was shown in rotation whenever the application was opened, and could be seen in the ‘advertising’ section in Greater Tokyo, recruiting a further 1442 participants for a total of 1657 valid respondents. All survey participants were notified they could win prizes of up to 80 USD by random lottery, as well as receive a survey results report. In total, 1335 (80.6%) of participants applied for the lottery incentive and 964 (58.2%) applied for the survey results.

Only respondents of 18 years or older, who self-reported as MSM (defined as identifying as ‘gay’ or ‘bisexual’, or having sexual experience with other men), and who provided online consent to participate in the survey were included in results. Ethics approval was granted by the University of Melbourne Human Research Ethics Committee, ID: 1646197. This research project was reviewed and approved by the Ministry of Foreign Affairs and the University of the Ryukyus in Japan to obtain the necessary visa approvals in order to undertake this study as a foreign researcher. The survey was an anonymous self-administered online questionnaire. Duplicate IP addresses were checked, and responses that did not sufficiently complete the questionnaire were removed. Participation was voluntary and there were no consequences for electing not to participate. All participants were provided with the details for local MSM helplines at the conclusion of the survey. The survey was translated into Japanese and back-translated into English by an independent Japanese native translator.

### Study measures

Socio-demographic characteristics of participants was determined by 12 items previously used in Japan [34], including age, gender, sexual orientation, marital status, birthplace, current residence, length of residence in Greater Tokyo, self-rated health, education, occupation, work hours, and intercourse partner sex. Gay mobile app use motivations were defined as ‘to find sex’, ‘to find friends, ‘to find a serious relationship’, or ‘to avoid being identified as gay’. Gay community participation measured years and frequency of gay bar and gay event attendance, frequency of gay bathhouse (*hattenba*) attendance, organized gay group activity participation in past six-months (defined as participation in a gay political or social meeting, community event such as a parade, party, fair, or volunteer activity), and identity as a gay community member. Respondents were asked three questions regarding HIV testing: ever having had an HIV test, having had an HIV test in the previous six months, and intention to get tested in the future (all with ‘yes’ or ‘no’ response options). ‘Recent testing’ was defined as testing within the previous six months. Respondents were asked frequency of lifetime condom use during penetrative or receptive anal intercourse with regular partners (defined as a boyfriend or committed romantic relationship), casual male partners (defined as a sex friend, ‘fuck buddy’, or any self-defined non-committed sexual relationship with a male partner), and sex with female partners. Lifetime condom use frequency was recorded as ‘never’, ‘rarely’, ‘most of the time’, and ‘always’. Following previous studies, low condom use was defined as ‘never use condoms’ or ‘rarely use condoms’, while high condom use was defined as ‘always use condoms’ and ‘use condoms most of the time’ [35]. Lifetime condom use analyses excluded those who did not have intercourse with each respective partner type.

### Statistical analysis

Data were analyzed using SPSS version 24. Descriptive statistics were used to describe all variables. We used multivariate binary regression analysis to examine the correlates of lifetime and recent HIV testing as well as future HIV testing intentions. The enter method was used and variables with unadjusted odds ratios yielding p-values of 0.05 or less were considered eligible for the multivariate model. Unadjusted and adjusted odds ratios with their respective 95% confidence intervals and p-values are reported in the tables.

## Results

### Socio-demographic characteristics

The socio-demographic characteristics of the sample are displayed in Table 1. Over half (53.4%) resided in the 23 wards of central Tokyo, and almost all (96.3%) respondents were born in Japan. The mean age was 35 and the vast majority were single (95.6%). They were well educated, with 58.1% completing university or a postgraduate degree. Nearly three-quarters (73.9%) were employed fulltime, and 4.2% were unemployed. There was high gay (85.1%) and bisexual (14.1%) identification, while 8.9% had both male and female sex partners. Just over half of participants had ever attended a gay bar (56.8%), just under half (47.6%) had ever attended a *hattenba* (gay bathhouse), and one-tenth (13.2%) participated in a gay group activity in the past six months.

**Table 1:** Socio-demographic characteristics.

		n	%
Current residence	Tokyo	883	53.4
	Greater Tokyo	553	33.4
	Another prefecture	209	12.6
	Another country	8	0.5
	Total	1653	100.0
Birthplace	Japan	1593	96.3
	Other	62	3.7
	Total	1655	100.0
Age	18–25	319	19.4
	26–35	550	33.4
	36–45	507	30.8
	46+	270	16.4
	Total	1646	100.0
Gender	Male	1641	99.2
	Other	14	0.8
	Total	1655	100.0
Marital status	No	1582	95.6
	Yes	72	4.4
	Total	1654	100.0
Occupation	Full-time	1133	68.5
	Part-time	185	11.2
	Student	168	10.2
	Self-employed	73	4.4
	Freelance	16	1.0
	Unemployed	69	4.2
	Other	9	0.5
	Total	1653	100.0
Education	High school or less	421	25.4
	Two-year technical school	274	16.5
	University	800	48.3
	M.A.	135	8.2
	PhD	26	1.6
	Total	1656	100.0
Intercourse partner sex	Men	1488	91.1
	Men and women	146	8.9
	Total	1634	100
Sexuality	Homosexual	1408	85.1
	Bisexual	233	14.1
	Other	14	0.8
	Total	1655	100
Health	Healthy	1056	63.9
	Fair or poor health	598	36.1
	Total	1654	100

### HIV prevention characteristics

Table 2 shows levels of HIV testing, lifetime condom use, and information sources. Almost three-quarters of respondents (72.8%) ever tested for HIV. Less than one-third (29.7%) had been tested in the past six months. However, the vast majority of the sample (88.2%) reported that they intended to obtain a test in the future. About one-third of those engaging in lifetime condomless anal intercourse (CLAI) with regular (31.5%) and casual (30.7%) male partners, and one-quarter engaging in condomless sex with female partners (23.6%), had been tested within the past six months. Among participants who had been tested for HIV in their lifetimes, over two-thirds (69.4%) engaged in CLAI with regular male partners, 54.1% engaged in CLAI with casual male partners, and 42.9% had condomless sex with female partners. Similarly, MSM who had never been tested for HIV engaged in risk-taking sexual behaviors: among participants who had never been tested, almost two-thirds (65.5%) engaged in CLAI with regular male partners, over half (54.8%) engaged in CLAI with casual male partners, and 44.4% had condomless sex with female partners. One-fifth (20%) of those never tested had no intention of undertaking future HIV testing. MSM aged 18–25 years had the lowest lifetime HIV testing (50%) and lowest rate of HIV testing in the past six-months (24.7%).

**Table 2:** HIV prevention characteristics.

		n	%
Have you ever been tested for HIV?	No	448	27.2
	Yes	1199	72.8
	Total	1647	100
Have you been tested for HIV in the last six months?	No	1148	70.3
	Yes	484	29.7
	Total	1632	100
Are you planning on getting tested for HIV in the future?	No	194	11.8
	Yes	1447	88.2
	Total	1641	100
How often do you use a condom with a regular (penetrative and receptive anal sex) male partner?^a^	No anal sex with regular male partner	246	14.9
	Never	217	15.5
	Rarely	302	21.5
	Most of the time	443	31.6
	Always	442	31.5
	Total	1404	100
How often do you use a condom with casual (penetrative and receptive anal sex) male partners?^b^	No anal sex with casual male partner	318	19.3
	Never	35	2.6
	Rarely	197	14.8
	Most of the time	490	36.8
	Always	609	45.8
	Total	1331	100
How often do you use a condom with female (penetrative and anal sex) partners?^c^	No sex with female partner	1358	82.6
	Never	29	10.1
	Rarely	28	9.8
	Most of the time	68	23.8
	Always	161	56.3
	Total	286	100
Whose advice about practicing safe sex would you be most likely to follow?	Doctor	630	38.3
	Regular sex partner	159	9.7
	Non-regular sex partner	20	1.2
	Internet resource	273	16.6
	Government agency	136	8.3
	LGBT Community Centre	418	25.4
	Friend	9	0.5
	Other	2	0.1
	Total	1647	100
HIV /AIDS information sources	School education	370	22.3
	Hospital pamphlet	329	19.9
	Gay internet resource	1441	87.0
	Government internet resource	255	15.4
	Friend	435	26.3
	Gay bar	316	19.1
	Television/newspaper	366	22.1
	Gay magazine	570	34.4
	Family	13	0.8
	Sex Partner	118	7.1
	Total	1656	100

^a^ analysis based on a subsample of 1404 people who have anal sex with a regular partner

^b^ analysis based on a subsample of 1331 people who have anal sex with a casual partner

^c^ analysis based on a subsample of 286 people who have sex with women

Gay internet resources were by far the leading source of HIV information, providing 87.0% of respondents with HIV/AIDS information. School education provided less than a quarter (22.3%) of respondents with HIV/AIDS information. Younger respondents were more likely to have been provided HIV/AIDS information at school, and half of respondents (49.5%) under 25, were provided with HIV/AIDS information in their school education compared to around a quarter (28.5%) of respondents aged 26–35, and less than 10% of respondents aged over 36. Despite gay internet resources providing the most HIV/AIDS information, respondents were most likely to follow a doctors’ advice about practicing safe sex (38.3%), followed by LGBT community centers (25.4%), compared to16.6% for gay internet resources. There was low trust in government agencies; less than one-tenth (8.3%) of respondents would follow a Japanese government agency’s advice about safe sex.

### Correlates of lifetime HIV testing

The prevalence of lifetime HIV testing and binary logistic regression models examining the correlates of lifetime HIV testing are presented in Table 3. There were 17 variables in the univariate regression with significant p-values of under 0.05. The final multivariate logistic regression model showed that the odds of having ever been tested for HIV were higher among MSM who were older, educated, HIV knowledgeable, out to close friends, who had ever attended a gay bar or event, *hattenba*, or participated in gay community activities in the past six-months, and had sex with regular or casual male partners. Lifetime HIV testing odds were lower among participants who: were students; were self-employed; were married; were residing outside of Greater Tokyo; were using gay mobile apps in order to avoid being identified as gay; also had female sex partners; and, who reported high condom use with regular male partners.

**Table 3:** Multivariate logistic regression for lifetime HIV testing.

	Number of respondents (n)	% Reporting Lifetime HIV Testing	Unadjusted Odds Ratio (95% C.I.)	p-Value	Adjusted Odds Ratio (95% C.I.)	p-Value
*Age (years)*						
18–25	316	52.2	REF			
26–35	550	74.0	1.60 (1.39–1.85)	0.000	1.37 (1.14–1.64)	0.001
36–45	506	80.4	1.55 (1.40–1.72)	0.000	1.43 (1.25–1.64)	0.000
46+	266	80.5	1.39 (1.26–1.52)	0.000	1.36 (1.21–1.54)	0.000
*Place of Birth*						
Japan	1584	72.8	0.99 (0.56–0.17)	0.969		
Other	62	72.6	REF			
*Education*						
High School or less	416	64.7	REF			
2 year university	272	72.8	1.10 (1.01–1.20)	0.026	1.02 (0.93–1.12)	0.724
University	799	75.3	1.67 (1.29–2.16)	0.000	1.44 (1.06–1.95)	0.020
Graduate degree	160	81.3	2.37 (1.52–3.7)	0.000	2.00 (1.20–3.33)	0.008
*Employment*						
Full-time work	1131	76.7	REF			
Part-time work	183	68.3	0.66 (0.47–0.92)	0.015	0.76 (0.51–1.13)	0.178
Student	167	50.3	0.31 (0.22–0.43)	0.000	0.63 (0.40–1.00)	0.048
Self-employed	96	69.8	0.70 (0.44–1.11)	0.129	0.52 (0.32–0.86)	0.010
Unemployed	68	79.4	1.17 (0.64–2.14)	0.607	1.29 (0.65–2.56)	0.468
*Current marital status*						
Single	1574	73.3	REF			
Married	72	61.1	0.57 (0.35–0.93)	0.024	0.51 (0.29–0.92)	0.025
*Current residence*						
Central Tokyo	880	76.9	REF			
Greater Tokyo	549	69.0	0.67 (0.52–0.85)	0.001	0.87 (0.67–1.14)	0.323
Other prefecture	207	63.8	0.53 (0.38–0.73)	0.000	0.67 (0.47–0.97)	0.034
*Intercourse partners*						
Only men	1480	75.1	REF			
Both men and women	146	56.2	0.43 (0.30–0.61)	0.000	0.90 (0.54–1.49)	0.688
*Health*						
Healthy	1052	72.1	0.89 (0.71–1.12)	0.311		
Fair/poor health	593	74.4	REF			
*Out to close friends*						
No	667	66.6	REF			
Yes	961	77.1	1.69 (1.36–2.11)	0.000	1.57 (1.21–2.03)	0.001
*Identify as a member of the gay community*						
No	1360	74.3	REF			
Yes	276	65.2	1.54 (1.17–2.03)	0.002	1.14 (0.83–1.56)	0.419
*Use gay mobile apps for sex*						
No	634	68.9	REF			
Yes	1006	75.3	1.38 (1.11–1.72)	0.004	1.05 (0.8–1.37)	0.729
*Use gay mobile apps to find friends*						
No	400	74.0	REF			
Yes	1232	72.6	0.93 (0.72–1.21)	0.596		
*Use gay mobile apps to avoid being identified as gay*						
No	1493	73.7	REF			
Yes	131	60.3	0.54 (0.37–0.78)	0.001	0.65 (0.43–1.00)	0.048
*Use gay mobile apps to find a serious relationship*						
No	763	74.8	REF			
Yes	866	71.1	0.83 (0.67–1.03)	0.094		
*Ever attended A gay bar*						
No	710	61.3	REF			
Yes	931	81.8	2.85 (2.28–3.57)	0.000	1.58 (1.21–2.05)	0.001
*Recent participation in gay group/community or volunteer activities*						
No	1425	70.9	REF			
Yes	218	85.3	2.38 (1.61–3.52)	0.000	1.59 (1.02–2.47)	0.040
*Hattenba attendance*						
No	858	64.9	REF			
Yes	783	81.5	2.38 (1.89–2.99)	0.000	1.53 (1.17–1.99)	0.002
			
*HIV Knowledge*: *Having other STIs doesn't increase chance of infection*						
Correct	1505	74.0	REF			
Incorrect	130	58.5	2.02 (1.40–2.92)	0.000	1.60 (1.04–2.45)	0.033
*Anal sex with regular male partners*						
No	244	57.4	REF			
Yes	1399	75.6	2.31 (1.74–3.05)	0.000	2.00 (1.39–2.86)	0.000
*Anal sex with casual male partners*						
No	317	57.0	REF			
Yes	1325	76.5	2.37 (1.84–3.06)	0.000	1.51 (1.08–2.13)	0.016
*Sex with female partners*						
No	1352	75.7	REF			
Yes	285	58.9	0.46 (0.35–0.60)	0.000	0.66 (0.45–0.99)	0.042
*Condom use with regular male partners*^a^						
Low	516	79.3	REF			
High	883	73.4	0.72 (0.56–0.94)	0.014	0.72 (0.54–0.96)	0.026
*Condom use with casual male partners*^b^						
Low	231	75.8	REF			
High	1094	76.6	1.05 (0.75–1.46)
*Condom use with female partners*^c^			
Low	56	55.4	REF
High	229	59.8	1.20 (0.67–2.17)

^a^ analysis based on a subsample of 1404 people who have anal sex with a regular partner; Low condom use = never/rarely use a condom; High condom use = most of the time/always use a condom

^b^ analysis based on a subsample of 1331 people who have anal sex with a casual partner; Low condom use = never/rarely use a condom; High condom use = most of the time/always use a condom

^c^ analysis based on a subsample of 286 people who have sex with women; Low condom use = never/rarely use a condom; High condom use = most of the time/always use a condom

### Correlates of recent HIV testing

The prevalence and univariate and multivariate logistic regression models examining the correlates of HIV testing in the past six-months are presented in Table 4. The final multivariate logistic regression model showed recent HIV testing odds were higher among MSM who attended *hattenba* (AOR, 1.30; 95% CI, 1.03–1.65), and who have a regular male partner (AOR, 1.85; 95% CI, 1.25–2.72).

**Table 4:** Multivariate logistic regression for recent HIV testing.

	Number of respondents (n)	% Reporting Recent HIV Testing	Unadjusted Odds Ratio (95% C.I.)	p-Value	Adjusted Odds Ratio (95% C.I.)	p-Value
*Age (years)*						
18–25	316	24.7	REF			
26–35	545	33.0	1.24 (1.06–1.45)	0.007	1.15 (0.96–1.39)	0.137
36–45	498	30.9	1.12 (1.01–1.24)	0.040	1.07 (0.94–1.22)	0.296
46+	264	26.9	1.04 (0.94–1.14)	0.474	1.00 (0.89–1.12)	0.979
*Place of Birth*						
Japan	1570	29.3	0.66 (0.39–1.11)	0.114		
Other	61	39.3	REF			
*Education*						
High School or less	415	26.7	REF			
2 year university	269	25.3	0.98 (0.90–1.07)	0.670	0.96 (0.87–1.05)	0.340
University	790	31.4	1.25 (0.96–1.63)	0.094	1.13 (0.85–1.51)	0.395
Graduate degree	158	36.1	1.55 (1.05–2.29)	0.029	1.40 (0.92–2.12)	0.120
*Employment*						
Full-time work	1115	31.3	REF			
Part-time work	182	28.0	0.85 (0.60–1.20)	0.358	0.87 (0.60–1.28)	0.484
Student	168	24.4	0.71 (0.49–1.02)	0.066	0.91 (0.57–1.45)	0.700
Self-employed	97	21.6	0.60 (0.37–0.99)	0.047	0.61 (0.36–1.02)	0.059
Unemployed	68	29.4	0.91 (0.53–1.56)	0.728	1.02 (0.58–1.79)	0.943
*Current marital status*						
Single	1560	29.7	REF			
Married	71	29.6	1.00 (0.59–1.68)	0.985		
*Current residence*						
Central Tokyo	871	32.5	REF			
Greater Tokyo	544	27.8	0.80 (0.63–1.01)	0.060	0.94 (0.73–1.20)	0.605
Other prefecture	206	22.3	0.60 (0.42–0.85)	0.005	0.69 (0.48–1.01)	0.056
*Intercourse partners*						
Only men	1468	31.0	REF			
Both men and women	143	19.6	0.55 (0.36–0.84)	0.005	0.68 (0.39–1.17)	0.165
*Health*						
Healthy	1045	29.8	1.01 (0.81–1.26)	0.936		
Fair/poor health	585	29.6	REF			
*Out to close friends*						
No	662	25.8	REF			
Yes	951	32.2	1.36 (1.09–1.70)	0.006	1.21 (0.95–1.53)	0.119
*Identify as a member of the gay community*						
No	274	25.9	REF			
Yes	1347	30.3	1.24 (0.93–1.67)	0.148		
*Use gay apps for sex*						
No	627	25.8	REF			
Yes	998	32.2	1.36 (1.09–1.70)	0.007	1.18 (0.92–1.50)	0.188
*Use gay apps to find friends*						
No	398	27.9	REF			
Yes	1219	30.3	1.12 (0.87–1.44)	0.367		
*Use gay apps to avoid being identified as gay*						
No	1497	30.1	REF			
Yes	130	26.2	0.82 (0.55–1.24)	0.348		
*Use gay apps to find a serious relationship*						
No	760	28.7	REF			
Yes	854	30.7	1.10 (0.89–1.36)	0.382		
*Ever attended A gay bar*						
No	704	24.6	REF			
Yes	922	33.6	1.56 (1.25–1.94)	0.000	1.19 (0.93–1.52)	0.174
*Recent participation in gay group/community or volunteer activities*						
No	1412	28.4	REF			
Yes	216	37.5	1.51 (1.12–2.04)	0.007	1.25 (0.90–1.72)	0.179
*Hattenba attendance*						
No	852	25.5	REF			
Yes	774	34.2	1.52 (1.23–1.89)	0.000	1.30 (1.03–1.65)	0.030
			
*HIV Knowledge*: *Having other STIs doesn't increase chance of infection*						
Correct	1493	30.2	REF			
Incorrect	128	23.4	1.41 (0.93–2.16)	0.109		
*Anal sex with regular male partners*						
No	242	17.8	REF			
Yes	1387	31.7	2.12 (1.50–3.00)	0.000	1.85 (1.25–2.72)	0.002
*Anal sex with casual male partners*						
No	313	21.4	REF			
Yes	1315	31.6	1.69 (1.27–2.27)	0.000	1.02 (0.72–1.45)	0.890
*Vaginal sex with female partners*						
No	1341	30.9	REF			
Yes	281	23.1	0.70 (0.50–0.90)	0.009	0.90 (0.60–1.33)	0.583
*Condom use with regular male partners*^a^						
Low	514	31.1	REF			
High	873	32.1	1.05 (0.83–1.32)	0.715		
*Condom use with casual male partners*^b^						
Low	227	30.8	REF			
High	1088	31.8	1.05 (0.77–1.43)	0.776		
*Condom use with female partners*^c^						
Low	56	21.4	REF			
High	225	23.6	1.13 (0.56–2.30)	0.736		

^a^ analysis based on a subsample of 1387 people who have anal sex with a regular partner; Low condom use = never/rarely use a condom; High condom use = most of the time/always use a condom

^b^ analysis based on a subsample of 1315 people who have anal sex with a casual partner; Low condom use = never/rarely use a condom; High condom use = most of the time/always use a condom

^c^ analysis based on a subsample of 281 people who have sex with women; Low condom use = never/rarely use a condom; High condom use = most of the time/always use a condom

### Correlates of future HIV testing intent

The prevalence and univariate and multivariate logistic regression models examining the correlates of future HIV testing intent are presented in Table 5. The final multivariate logistic regression model showed future HIV testing intention odds were higher among MSM who attend gay bars (AOR, 1.47; 95% CI, 1.06–2.05), use gay mobile apps to find friends (AOR 1.58; 95% CI, 1.12–2.22), are healthy (AOR 1.46; 95% CI, 1.06–2.03), have a regular sex partner (AOR, 2.01; 95% CI, 1.31–3.10), and who engage in high condom use with casual male partners (AOR, 2.09; 95% CI, 1.38–3.18). Lower odds of intention of future HIV testing was associated with respondents who were married (AOR, 0.45; 95% CI, 0.24–0.85), and attended *hattenba* (AOR, 0.64; 95% CI, 0.46–0.90).

**Table 5:** Multivariate logistic regression for future HIV testing intent.

	Number of respondents (n)	% Reporting Future HIV Testing Intent	Unadjusted Odds Ratio (95% C.I.)	p-Value	Adjusted Odds Ratio (95% C.I.)	p-Value
*Age (years)*						
18–25	318	90.6	REF			
26–35	546	90.1	0.98 (0.77–1.23)	0.843	1.06 (0.80–1.40)	0.682
36–45	503	87.3	0.90 (0.77–1.04)	0.153	0.97 (0.81–1.17)	0.770
46+	265	83.0	0.85 (0.75–0.96)	0.007	0.93 (0.80–1.08)	0.327
*Place of Birth*						
Japan	1579	88.0	0.65 (0.256–1.63)	0.354		
Other	61	91.8	REF			
*Education*						
High School or less	418	86.1	REF			
2 year university	270	87.4	1.03 (0.92–1.15)	0.629	1.04 (0.92–1.18)	0.495
University	793	88.5	1.24 (0.87–1.77)	0.227	1.21 (0.82–1.79)	0.341
Graduate degree	160	93.1	2.18 (1.11–4.27)	0.023	2.01 (1.00–4.07)	0.051
*Employment*						
Full-time work	1122	88.1	REF			
Part-time work	185	85.4	0.79 (0.50–1.23)	0.289	0.94 (0.57–1.55)	0.797
Student	167	94.0	2.11 (1.08–4.10)	0.028	1.78 (0.81–3.92)	0.152
Self-employed	97	84.5	0.73 (0.41–1.31)	0.295	0.77 (0.42–1.41)	0.393
Unemployed	68	86.8	0.88 (0.43–1.82)	0.729	1.14 (0.53–2.45)	0.746
*Current marital status*						
Single	1568	88.8	REF			
Married	72	73.6	0.35 (0.20–0.60)	0.000	0.45 (0.24–0.85)	0.013
*Current residence*						
Central Tokyo	877	87.5	REF			
Greater Tokyo	547	90.1	1.30 (0.92–1.84)	0.131		
Other prefecture	206	85.4	0.84 (0.54–1.23)	0.427		
*Intercourse partners*						
Only men	1475	88.7	REF			
Both men and women	146	89.7	1.13 (0.65–2.00)	0.677		
*Health*						
Healthy	1048	90.0	1.57 (1.16–2.13)	0.003	1.46 (1.06–2.03)	0.022
Fair/poor health	591	85.1	REF			
*Out to close friends*						
No	661	87.6	REF			
Yes	961	88.6	1.10 (0.81–1.49)	0.557		
*Identify as a member of the gay community*						
No	272	88.6	REF			
Yes	1358	88.1	0.95 (0.63–1.43)	0.804		
*Use gay apps for sex*						
No	553	87.6	REF			
Yes	889	88.6	1.10 (0.81–1.50)	0.543		
*Use gay apps to find friends*						
No	398	83.9	REF			
Yes	1228	89.7	1.68 (1.21–2.32)	0.002	1.58 (1.12–2.22)	0.009
*Use gay apps to avoid being identified as gay*						
No	1487	88.2	REF			
Yes	131	88.5	1.04 (0.59–1.82)	0.896		
*Use gay apps to find a serious relationship*						
No	763	86.2	REF			
Yes	860	90.1	1.46 (1.07–1.97)	0.016	1.15 (0.83–1.59)	0.402
*Ever attended A gay bar*						
No	704	86.1	REF			
Yes	931	89.8	1.42 (1.05–1.92)	0.022	1.47 (1.06–2.05)	0.022
*Recent participation in gay group/community or volunteer activities*						
No	1420	88.0	REF			
Yes	217	88.9	1.09 (0.70–1.72)	0.699		
*Hattenba attendance*						
No	885	90.2	REF			
Yes	780	86.0	0.67 (0.50–0.91)	0.010	0.64 (0.46–0.90)	0.011
			
*HIV Knowledge*: *Having other STIs doesn't increase chance of infection*						
Correct	1500	88.1	REF			
Incorrect	129	89.9	0.83 (0.46–1.51)	0.545		
*Anal sex with regular male partners*						
No	243	79.0	REF			
Yes	1395	89.7	2.29 (1.61–3.26)	0.000	2.01 (1.31–3.10)	0.001
*Anal sex with casual male partners*						
No	315	83.5	REF			
Yes	1322	89.3	1.62 (1.15–2.28)	0.006	1.32 (0.85–2.06)	0.211
*Vaginal sex with female partners*						
No	1347	88.4	REF			
Yes	285	86.7	0.85 (0.58–1.24)	0.385		
*Condom use with regular male partners*^a^						
Low	516	90.1	REF			
High	879	89.5	0.94 (0.65–1.35)	0.729		
*Condom use with casual male partners*^b^						
Low	230	80.4	REF			
High	1092	91.1	2.50 (1.70–3.67)	0.000	2.09 (1.38–3.18)	0.001
*Condom use with female partners*^c^						
Low	57	80.7	REF			
High	228	88.2	1.78 (0.82–3.85)	0.143		

^a^ analysis based on a subsample of 1395 people who have anal sex with a regular partner; Low condom use = never/rarely use a condom; High condom use = most of the time/always use a condom

^b^ analysis based on a subsample of 1322 people who have anal sex with a casual partner; Low condom use = never/rarely use a condom; High condom use = most of the time/always use a condom

^c^ analysis based on a subsample of 285 people who have sex with women; Low condom use = never/rarely use a condom; High condom use = most of the time/always use a condom

## Discussion

This survey shows that gay mobile app users in Greater Tokyo are unlikely to reach the first of the 90-90-90 aims set by UNAIDS, having 90% of people living with HIV (PLHIV) diagnosed by 2020, with 72.8% of MSM respondents ever tested for HIV. Two-fifths (40.4%) of MSM who had undergone lifetime HIV testing underwent testing at least once every six months, meeting CDC MSM yearly HIV-testing recommendations [36]. Comparisons with existing Japanese data show lifetime testing was similar to previous MSM lifetime testing reports. For example, a 2013 survey of 491 MSM in Greater Tokyo found almost three quarters (72.9%) to have been tested for HIV in their lifetime, over a third (35.0%) in the past year, and almost nine-tenths (86.2%) had considered getting an HIV test [10]. Lifetime testing was higher than previous data collected by MSM internet surveys in Asia, including Korea [37], China (60.5% [18]; 58.3% [38]) and Vietnam (23.5%; [39]), possibly due to higher availability in Japan of free HIV testing, counselling, antiretroviral therapy, and lower stigma, associated with HIV testing uptake. Respondents had higher lifetime testing in this research than gay bar attending MSM in Japan with a similar age profile to this study (61.0%; Shiono [9]), supported by previous findings where gay mobile app users were also found to have higher lifetime testing than non-users in China [18] and meta-analysis [29].

Socio-demographic factors significantly associated with lifetime testing (age, education, residence) were similar to previous findings among both gay mobile app using and non-using MSM in Japan and abroad. Higher education was associated with higher lifetime testing among respondents similar to previous findings among venue-attending MSM in Japan [9], and abroad [38,40,41], and rural residency was significantly associated with lower lifetime HIV testing among MSM, possibly due to lower HIV information access and lower tolerance for sexual minorities [42], and lower HIV knowledge [43]. Lifetime HIV testing was significantly lower among MSM youth, and only half (52.2%) of respondents aged 18–25 had been tested for HIV, and one-quarter (24.7%) in the past 6-months. Youth have been found to have lower HIV testing among MSM who use gay mobile apps [21], internet [44] and attend gay venues [40]. Youth may have lower lifetime HIV testing because they are younger, and therefore have had fewer partners, thus perceive their risk as lower, and have had fewer years to be tested [45]. However, studies have shown that compared to older MSM, young MSM (YMSM) are more likely to engage in CLAI concurrently with both their primary partner and with someone outside the relationship [46], and are thus at higher risk for HIV [47]. Only half of respondents aged 18–25 had learned about HIV in schools, indicating the pressing need for good quality sex education in Japanese high schools.

Respondents who engaged in anal sex with regular partners were twice as likely to have been tested in their lifetime, and more likely to have been tested recently. Although due to the cross-sectional nature of this study causation cannot be determined, two explanations are possible for the association between anal intercourse with male partners and lifetime and recent HIV testing. First, perceived risky sexual behaviors may lead to HIV testing. Thus, MSM who have engaged in risky sexual behaviors with regular and casual male partners may perceive this risk and get tested. A second potential explanation is that respondents are engaging in negotiated safety, where regular partners agree not to have CLAI with outside partners after being tested with regular partners [48]. HIV negative seroconcordant relationships (e.g. where both partners are HIV negative), are associated with low HIV incidence [49]. However, respondents not discussing or complying with negotiated safety is a risk factor for HIV transmission as MSM often continue to use gay mobile apps throughout relationships [23], and over half were found to have both regular and casual partners concurrently [50]. Condom use may become increasingly inconsistent as casual MSM relationships continue [51], posing significant risk of infection to regular partners with whom they have a higher number of sex acts, more frequent receptive roles, and lower condom use; subsequently, regular partners accounted for 68% of MSM HIV transmissions in the US [52], but only one-tenth in Australia where negotiated safety has been promoted more among MSM [53].

Although respondents who engaged in CLAI with regular male partners were more likely to have undergone testing in their lifetime, participants who engaged in CLAI with casual male partners or condomless sex with female partners were not more likely to have undergone lifetime or recent testing. Without knowing an individual’s Pre-exposure prophylaxis (PrEP) status and viral load, it is difficult to ascertain the risk posed by CLAI. However, PrEP is not currently approved for HIV prevention in Japan [54], and MSM engaging in CLAI with casual male partners may therefore be at higher risk of HIV but are not following CDC testing guidelines for high-risk individuals to undergo testing more regularly [36].

Unlike anal intercourse with male partners, sex with female partners was significantly associated with lower lifetime HIV testing. Previous research has found lower regular and lifetime HIV testing associated with both bisexual behavior [40] and bisexual identity [9,45,55] among MSM. Increased internalized homo-negativity, associated with lower HIV testing [56], and lower gay community attachment may potentially explain the association between MSM who have intercourse with both male and female partners and lower lifetime HIV testing. All forms of gay community attachment and participation (gay bar attendance, gay community activity participation, *hattenba* attendance, as well as coming out to close friends) were associated with higher lifetime testing. Lower gay community attachment was associated with less access to HIV testing information, and lower emotional support for a positive HIV result without which the fear of a positive result may make MSM reticent of being tested [45]. More explicitly, in Japan, free HIV testing facilities and HIV prevention information is almost exclusively advertised through gay NGOs at gay venues. Survey respondents with both male and female sex partners participated less in the gay community, and therefore had less information regarding access to HIV testing facilities.

### Increasing access to HIV testing

In order to increase testing numbers, both HIV testing promotion and additional testing strategies such as expansion of rapid voluntary counselling and testing (VCT), which is associated with a threefold increase in HIV testing uptake and a twofold increase in receipt of results [57], and approval of self-testing kits such as rapid, oral, fluid HIV home tests, a cost-efficient and effective prevention method for networks with high-risk sexual practices [58] should be examined. Utilizing the ubiquity of vending machines in Japan in order to sell self-testing kits together with other items such as in China [59], particularly outside of urban areas such as Tokyo where access to testing is poor, may lead to an increase in testing if promoted effectively. Research into the kinds of testing services Japanese MSM, particularly non-community attached MSM, would be likely to utilize may also give useful feedback for appropriate resource allocation for HIV testing among high-risk populations. Previous research has shown significantly higher HIV testing levels after HIV testing promotions using mobile apps [60]. Increasing HIV testing rates for non-gay community MSM, specifically bisexual and young MSM may help stop HIV bridging into the Japanese heterosexual population, which has been previously identified as possible but low risk [61]. Gay mobile app users are largely willing to receive HIV prevention materials via mobile apps [20], and the majority of respondents requested results from this study, showing an interest in prevention and MSM community behaviors. Because respondents use gay mobile apps frequently, and Japanese gay mobile apps have shown willingness to provide price discounts for MSM surveillance and prevention research, utilization of popular gay mobile apps to promote HIV testing facilities may prove to be a cost-effective prevention policy for targeting non-community attached MSM youth who are particularly at-risk for HIV infection and increasingly use gay mobile apps instead of attending traditional gay venues to meet sex partners in Japan [25]. In order to facilitate testing, increasing the amount of free testing facilities, extending testing hours in order to fit around busy Japanese work schedules, adding free optional HIV testing to the annual health check-ups available to all residents of Japan (with proper privacy protection), and providing cultural competency training for interviewers and health providers at testing centers should be implemented.

### Limitations

The research was subject to a variety of limitations that must be taken into account. Respondents were recruited through an advertisement, and may not be representative of all Japanese MSM. Furthermore, the behaviors and attitudes reported were self-rated and may have been subject to social desirability bias where male participants may downplay certain behaviors they believe to be undesirable [62]. However, because the survey was anonymous and online this effect was likely minimized. Due to limited resources, this research only used specific gay mobile applications. HIV-positive individuals were not excluded from this study. Recent HIV testing, and future HIV testing intention may not be representative of the reality of the situation in Japan. However, the levels of recent HIV testing and future HIV testing intention reported in this study were similar to previous findings in Japan. Other mobile SNS applications are specifically used by particular MSM populations (such as “Mister” for older men), and may contain different MSM populations worthy of future research. All of these apps function similarly, and this study method would likely be appropriate for future comparative and inclusive research in Japan.

## Conclusion

These results show that MSM who use gay mobile apps in Greater Tokyo do not meet the CDC yearly testing recommendations for high risk populations. Although higher than neighbouring nations, HIV testing was still infrequent compared to other countries providing similar levels of care. Considering limited HIV prevention funding in Japan for MSM, moderate lifetime and recent testing, and the large number of gay mobile app users, utilization of popular gay mobile apps to promote nearby HIV testing facilities may be an effective prevention policy to target non-community attached MSM, particularly at-risk youth and individuals at risk for sudden-onset AIDS.
